# Hypoxic NO-donor nitrite protects sGC-dependently against morbidity and mortality associated with sterile inflammatory shock in mice

**DOI:** 10.1186/cc9677

**Published:** 2011-03-11

**Authors:** A Cauwels, B Vandendriessche, E Rogge, S Shiva, P Brouckaert

**Affiliations:** 1 UGent & VIB, Gent, Belgium; 2 University of Pittsburgh, PA, USA

## Introduction

For a long time nitrite (NO_2_^-^) was believed to be an inert metabolite of the endogenous vasodilator NO. Recently, however, nitrite was identified as an important biologic NO reservoir in vasculature and tissues, contributing to hypoxic signaling, vasodilation and cytoprotection after ischemia-reperfusion injury. Reduction of nitrite to NO may occur enzymatically at low pH and oxygen tension by deoxyhemoglobin or deoxymyoglobin, xanthine oxidase, mitochondria or NO synthase. Considering that NO may exert protective effects in inflammatory and septic shock, and that circulating nitrite may function as a source of NO in hypoxic and/or acidic conditions present in ischemic microvasculature of vital organs during shock, we decided to test the protective capacity of nitrite on toxicity associated with inflammatory shock.

## Methods

We studied sterile models of shock (induced by intravenous TNF or LPS) and a septic CLP model in female C57Bl/6 mice. NaNO_2 _treatments were done intravenously. To monitor morbidity, rectal body temperatures were measured and mortality was recorded. In addition, mice were sacrificed 2 or 6 hours after challenge to analyze serum markers for organ damage, as well as mitochondrial parameters, ATP production and infiltration of myeloid cells. Hemodynamic parameters were determined in conscious mice via radiotelemetry, using PA-C10 probes (Data Sciences International).

## Results

Low doses of nitrite significantly ameliorated hypothermia, organ damage and mortality induced by a lethal TNF challenge. Mechanistically, nitrite-dependent protection was associated with improved mitochondrial functioning, demonstrated by complex I, complex IV and aconitase activities in the liver and heart. In addition, nitrite protection was largely abolished in mice deficient for the α_1_-subunit of soluble guanylate cyclase (sGCα_1_), one of the principle intracellular NO receptors and signal transducers in the cardiovasculature. Interestingly, nitrite delayed and attenuated TNF-induced bradycardia and hypotension as well. In addition, higher doses of nitrite could also protect against toxicity induced by Gram-negative LPS, but not against mortality induced by CLP.

## Conclusions

We show that nitrite can protect against mitochondrial and organ damage in inflammatory sterile shock via sGC-dependent signaling. This may include hypoxic vasodilation, necessary to maintain microcirculation and organ function, as well as cardioprotection.

